# Uneven burden of cardiac amyloidosis in people of African descent — global imbalance in resources and access

**DOI:** 10.1186/s44263-023-00016-3

**Published:** 2023-09-01

**Authors:** Ernest C. Madu, Kenechukwu Mezue

**Affiliations:** 1 Division of Cardiovascular Medicine, Heart Institute of the Caribbean and HIC Heart Hospital, Kingston, Jamaica; 2 Section of Cardiovascular Medicine, Yale School of Medicine, New Haven, CT USA

**Keywords:** Cardiac Amyloidosis, African descent, Heart Failure, Global health inequality

## Abstract

Transthyretin cardiac amyloidosis (TTR-CA) is now increasingly becoming recognized as an important cause of heart failure, and some studies have shown that as much as a third of diastolic heart failure could be attributed to TTR-CA. Black populations are particularly at risk for TTR-CA as the most common form of the disease (hereditary TTR-CA) has a genetic basis and the gene responsible is most prevalent among people with West African ancestry. This perspective piece explores the challenges that individuals of African and Caribbean populations face when confronted with the burden of TTR-CA. Key issues include the absence of rigorous disease registries, deficits in human resources, a lack of infrastructure for testing and treatment, poor awareness and health literacy, financial limitations including an inadequate public health budget, and the absence of social safety nets. To address these challenges, proactive strategies are needed to build infrastructure and local capacity which will provide the framework for an effective response. Interventions should include healthcare financing mechanisms to protect and care for vulnerable and at-risk populations with a long-term strategy of increasing the financial remuneration for health workers in developing countries to prevent the brain drain. Additionally, pharmaceutical companies need to play an active role in promoting inclusive access and global health equity in the access to the new treatments for TTR-CA which predominantly affects Black populations. Collaborative ventures with international centers of excellence can help improve access in these communities, leveraging their expertise and resources.

## Background

Transthyretin cardiac amyloidosis (TTR-CA) was once thought to be a rare cause of heart failure, but it is now increasingly recognized as an important cause of heart failure, accounting for nearly a third of diastolic heart failure in some populations [1–3]. The pathogenesis of TTR-CA is linked to the deposition of monomers of transthyretin (previously known as prealbumin) in the cardiac extracellular space. This leads to stiff ventricles with a restrictive cardiomyopathy phenotype, eventually leading to congestive heart failure, atrial fibrillation, and cardiac death [4, 5]. TTR-CA has two main forms: a wild type (wtTTR) and a hereditary type (hTTR). The most common hereditary form is related to a single gene mutation (V142I) prevalent in people of West African descent. Cardiac amyloidosis, specifically transthyretin cardiac amyloidosis (TTR-CA), poses a significant health burden in communities of African descent. Despite the prevalence of this condition in African populations, there remains a lack of reliable data on the phenotypes of TTR-CA within African and Caribbean communities, hindering effective action.

This perspective explores the multiple factors contributing to the global imbalance in resources and access for individuals of African descent affected by TTR-CA. Key issues include the absence of rigorous documentation, deficits in trained manpower and resources, lack of infrastructure for testing and treatment, affordability and access challenges, limited and dwindling local capacity for research and training, health literacy and cultural inhibitions, inadequate public health budget and infrastructure, political will, economic costs and implications, the absence of social safety nets, and the intersectionality of these variables affecting recognition and management of TTR-CA.

To address these challenges, proactive strategies are needed to build infrastructure and capacity to provide the framework for an effective response. Healthcare financing mechanisms must be established to protect and care for vulnerable and at-risk populations. Deliberate poaching of human capital from underdeveloped economies encourages brain drain and undermines the ability of low-resource nations to develop meaningful local capacity. Additionally, the role of pharmaceutical companies in ensuring inclusive access and health equity, while recognizing economic challenges in low- and middle-income countries with predominantly Black populations, must be examined. Collaborative ventures with international centers of excellence can help improve access in these communities, leveraging their expertise and resources.

## A genetic basis for cardiac amyloidosis

While it can affect individuals of any ethnic background, there is growing evidence that people of African descent are disproportionately affected by cardiac amyloidosis, particularly transthyretin cardiac amyloidosis (TTR-CA) [6–10]. This condition has a significant impact on the affected individuals and their communities, leading to reduced quality of life, increased morbidity and mortality rates, and a higher burden on healthcare systems. Understanding the unique characteristics and challenges of TTR-CA in individuals of African descent is crucial for developing targeted interventions and improving outcomes in these populations.

**Fig. 1:**
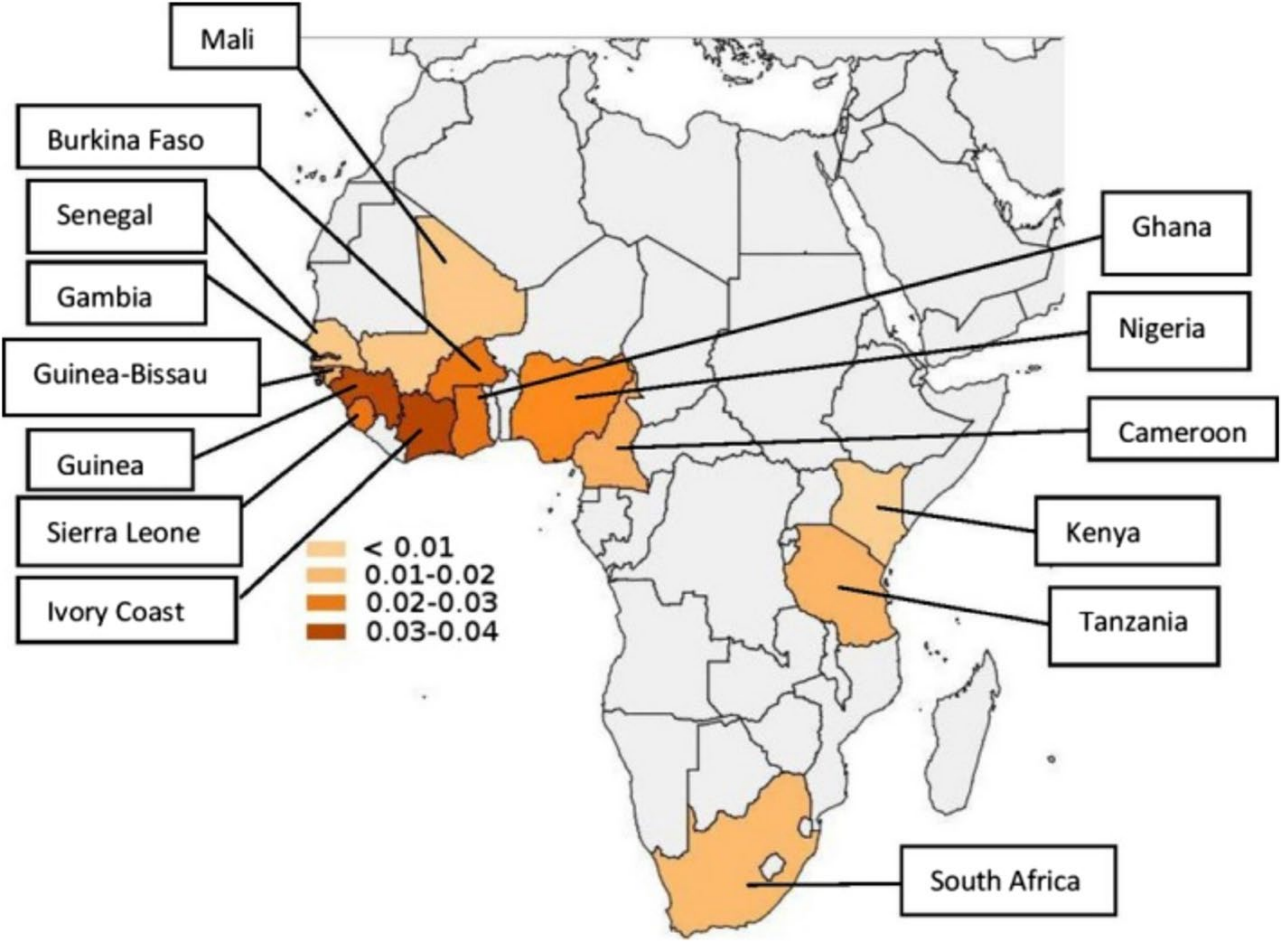
Distribution of the CA-TTR V122I allele (V142I allele) in Africa. Legend: The allele frequencies are indicated by color in the figure. Populations from the countries colored light gray were not analyzed (from: Jacobson, D. R., Alexander, A. A., Tagoe, C., Garvey, W. T., Williams, S. M., Tishkoff, S., Modiano, D., Sirima, S. B., Kalidi, I., Toure, A., & Buxbaum, J. N. (2016). The prevalence and distribution of the amyloidogenic transthyretin (TTR) V122I allele in Africa. *Molecular Genetics & Genomic Medicine*, *4*(5), 548–556. https://doi.org/10.1002/mgg3.231)

A genetic basis for cardiac amyloidosis was first suggested by the Los Angeles County study where investigators reviewed 52,370 autopsies and found that the prevalence of cardiac amyloidosis among Black Americans (1.6%) was significantly greater than the prevalence among White Americans (0.42%), even though all other types of amyloidosis were less prevalent among Black Americans [11]. Eventually, more than 150 point mutations of the transthyretin gene have been discovered [12]. The most common mutation results from a G to A transition at a CG dinucleotide in codon 142 (122 of the mature protein) which causes an isoleucine substitution for valine (V142I) [13]. This mutation is almost exclusively found in people of African descent [5, 14]. Cohort studies in the USA have shown that 3–4% of self-identified Black Americans carry this gene variant (allele frequency 0.02), and it is rare in people of other ethnicities [7–9, 15–17]. The only genetic study in Africa has shown that this variant allele is most prevalent (allele frequency 0.0253, about 5% of the population) in the contiguous West African countries of Sierra Leone, Guinea, Ivory Coast, Burkina Faso, Ghana, and Nigeria (see Fig. 1) [18].

## Diagnosis and treatment of hereditary cardiac amyloidosis

In the past, an endomyocardial biopsy was required to diagnose hTTR-CA definitively; however, this could only be done in large tertiary centers, and the procedure required advanced training and carried a substantial risk of serious adverse events such as right ventricular perforation. Recently, a simpler nuclear imaging scan (pyrophosphate scan) has been shown to be 99% sensitive at detecting hTTR-CA and is now the diagnostic test of choice [19, 20]. Other modalities, such as cardiac magnetic resonance imaging and echocardiographic strain imaging, can play a role in diagnosing hTTR-CA, even though these techniques have lower diagnostic yield.

A simple screening and diagnostic strategy proposed by the European Society for Cardiology’s Working Group on cardiac amyloidosis suggest that patients with left ventricular wall thickness greater than or equal to 12 mm plus one or more “red flag” features (new aortic stenosis, hypotension/normotension if previously hypertensive, autonomic and sensory dysfunction, peripheral neuropathy, proteinuria, skin bruising, bilateral carpal tunnel syndrome, ruptured biceps tendon, decreased voltage/conduction disease/pseudo Q waves on the electrocardiogram (ECG)) should undergo subsequent noninvasive testing with technetium-99 m pyrophosphate scintigraphy (PYP scan), echocardiography, or cardiac magnetic resonance imaging for diagnosis [21]. Diagnostic pathways such as heart failure pathways (for example, a new diagnosis of heart failure) and imaging pathways (for example, an incidental finding of left ventricular hypertrophy or increased wall thickness on a routine scan) should trigger off a cardiac amyloidosis workup [22, 23]. Such a strategy will ensure early diagnosis which is key to reducing the burden of the disease in the community [24].

In the last decade, several strides have been made in discovering treatment options for the disease that hold a lot of promise. Tafamidis is an oral medication that binds to transthyretin, stabilizes the transthyretin tetramer, and prevents the dissociation of the tetramer into monomers and subsequent amyloid deposition in the tissues [25]. In a multicenter, international, double-blind, placebo-controlled, phase 3 trial involving 441 patients and 30 months of follow-up, tafamidis demonstrated a statistically significant 30% reduction in all-cause mortality compared to placebo [25].

There are other potentially effective treatments on the horizon. In the last decade, gene editing and ribonucleic acid (RNA) interference techniques have been successfully employed in early-stage studies to suppress the production of transthyretin in the liver [26, 27]. Phase 3 trials utilizing these techniques are expected in the coming years. Very recently, a recombinant human antibody was shown to reduce cardiac tracer uptake on nuclear scintigraphy and extracellular volume on cardiac magnetic resonance imaging (both of which are imaging-based surrogate markers of cardiac amyloid load) over 12 months compared to placebo [28].

## A significant cause of heart failure in African Americans

Several cohort studies have shown that carriers of the gene variant (both heterozygous and homozygous, given the autosomal dominant mode of inheritance) have a higher incidence of heart failure and heart failure hospitalizations [7, 9, 15, 17]. Older cohorts show an increased incidence of heart failure and all-cause mortality [8, 29]. Heart failure is a significant global health concern with an increasing prevalence and burden, particularly among people of African descent. In US cohorts, the age-adjusted heart failure mortality rate in 2017 was nearly three times higher in young Black Americans than in young White Americans [30]. In addition, heart failure hospitalizations were more than twice higher for Black Americans than White Americans [31, 32].

Even though social determinants of health contribute to these disparities, it is also likely that a lack of attention to this distinct phenotype of heart failure undoubtedly contributes to the burden of disease in this minority population. In the USA alone, there are estimated to be at least 1.6 million carriers of the common V142I hTTR mutation, and even given a conservatively estimated low penetrance of 10–20%, it means that there may be 160,000 to 320,000 Black Americans at risk of early heart failure who are relatively underdiagnosed [33]. A recent study showed that Black Americans with cardiac amyloidosis tend to be diagnosed much later (and in reduced ejection heart failure) than their Caucasian counterparts with worse mortality on follow-up analysis [34]. Clinical pathways as suggested in the previous section could play a role in ensuring earlier diagnosis in this cohort [6].

Another important consideration is that the treatment of heart failure with cardiac amyloidosis is slightly different from usual heart failure treatment. hTTR-CA patients do not tolerate or respond to standard guideline-directed medical therapy medications such as beta-blockers and angiotensin receptor blockers/neprilysin inhibitors as these cause significant hypotension in hTTR-CA patients and could indeed be harmful [35]. This adds to these patients’ disease burden when underdiagnosed in the community.

## An uncharted burden in West Africa and the African diaspora

It is established that the mutant transthyretin gene arose from the West African subregion, and the allele frequency for the mutation is highest in that region (Fig. 1) [18]. However, there is still a paucity of reliable data regarding the clinical impact of hTTR-CA in West African populations. There is likely severe underdiagnosis of amyloidosis in West Africa and the African diaspora, as there is limited access to diagnostic testing due to financial and technological constraints in these low- and middle-income countries.

The epidemiology of heart failure in this population needs further study to address this underdiagnosis and correct some myths associated with the etiology of heart failure in the African diaspora. For example, the predominance of “hypertensive heart disease” as the purported major cause of heart failure in West Africa is likely based on visualizing enlarged left ventricular walls on two-dimensional echocardiography in patients with a history of hypertension and normal ECG patterns or generous voltage patterns on the ECG (as amyloidosis is well known to cause low-voltage complexes). However, Dungu et al. showed that 44% of patients with hTTR-CA had normal ECGs with normal voltage complexes, with 25% meeting left ventricular hypertrophy criteria [36]. Also, a case series of autopsies of patients with sudden cardiac death in Western Nigeria reports that hypertensive heart disease was the predominant cause of death, even though no Congo red staining was performed on those patients, which could indeed reveal undiagnosed hTTR-CA [37].

## Critical impediments

One of the critical issues in addressing TTR-CA in communities of African and Caribbean descent is the absence of reliable data on the phenotypes of the disease in these populations. This lack of data hinders the understanding of disease progression, response to treatment, and overall burden of TTR-CA. Rigorous documentation of TTR-CA cases and comprehensive clinical data collection is essential to guide action and improve outcomes. Accurate data can help identify risk factors, inform screening and diagnostic strategies, and guide the development of targeted interventions that are tailored to the unique characteristics of TTR-CA in African and Caribbean communities. Systematic data collection also facilitates epidemiological studies, which can help identify patterns, risk factors, and potential interventions to mitigate the burden of TTR-CA. Furthermore, robust documentation can provide support for advocacy efforts, raise awareness about the disease, and encourage resource allocation for research, infrastructure, and healthcare services.

The deficit in trained workforce and human resources is a significant problem in many African countries and communities. For example, in 2019, Nigeria, a country of about 250 million people, had 3.67 physicians per 10,000 population. A neighboring country, Niger, had 0.38 physicians per 10,000 population, while the USA, a developed country, had 35.18 physicians per 10,000 population, which is a 100-fold higher density of physicians [38]. Addressing TTR-CA in communities of African descent is impeded by a deficit of trained healthcare professionals and limited resources. Healthcare providers need specialized training to diagnose and manage TTR-CA effectively. However, there is a scarcity of experts with knowledge and experience in the diagnosis, treatment, and ongoing care of this condition. This is further exacerbated by the deliberate actions of more developed economies who go to great lengths to poach and recruit the most highly trained and skilled healthcare workers from already significantly depleted and overburdened low-resource economies. To overcome this challenge, efforts should be directed towards expanding training programs, providing educational resources, improving working conditions, and establishing mentorship initiatives to enhance the expertise of healthcare professionals in TTR-CA management.

A lack of infrastructure for the diagnosis and treatment of TTR-CA is yet another major challenge and hampers efforts at addressing TTR-CA in African and Caribbean communities. Access to advanced diagnostic tools, such as genetic testing and specialized imaging modalities like nuclear imaging, cardiac magnetic resonance imaging, and advanced echocardiographic strain imaging, is essential for the accurate and timely diagnosis of TTR-CA. However, many healthcare facilities in African and Caribbean communities lack the necessary equipment and expertise to perform these tests. For example, Ghana (a country of about 32 million people in West Africa) has only one single-photon emission computed tomography (SPECT) system for the whole country [39]. The program was established in 2005 but only started a cardiac SPECT program in May 2023 with the assistance of the Society of Nuclear Medicine and Molecular Imaging (SNMMI) [40]. Such international collaborations are vital to bridging the infrastructure gap.

Furthermore, access to specialized treatments, such as transthyretin stabilizers or gene-silencing therapies, is limited. Investing in infrastructure development, including establishing cardiac centers of excellence, improving access to advanced diagnostic technologies, and ensuring the availability of appropriate treatment options, is crucial for improving the management of TTR-CA in these populations.

Challenges related to affordability and access significantly impact the recognition and management of TTR-CA in communities of African descent. Many individuals in these communities face financial constraints that hinder their ability to seek healthcare services, undergo diagnostic tests, and afford the high-cost treatments associated with TTR-CA. Additionally, limited access to healthcare facilities, particularly in rural or underserved areas, further exacerbates the problem. Efforts should focus on improving financial assistance programs, expanding insurance coverage, and implementing alternative healthcare financing mechanisms and community-based initiatives to increase access to affordable healthcare services and reduce financial barriers for individuals at risk of or affected by TTR-CA.

Health literacy and cultural factors play a significant role in the access to information, healthcare-seeking behaviors, and disease management in communities of African descent [41, 42]. Limited health literacy can hinder individuals’ understanding of TTR-CA symptoms, risk factors, and the importance of seeking timely medical attention. Cultural beliefs, stigma, and a mistrust of the healthcare system may also influence healthcare-seeking behaviors and adherence to treatment regimens. To address these challenges, culturally sensitive educational campaigns, community engagement, and the involvement of trusted community leaders and organizations are necessary to improve health literacy, dispel misconceptions, and foster a supportive environment for individuals affected by TTR-CA.

The low public health budget and weak public health infrastructure are also a significant hurdle in many communities of African descent. Insufficient funding limits the implementation of comprehensive public health programs, including awareness campaigns, screening initiatives, and research efforts. Weak infrastructure, including inadequate healthcare facilities, a limited healthcare workforce, and fragmented and asymmetric healthcare systems, undermines the delivery of timely and quality care for individuals with TTR-CA. Total healthcare spending per capita (in purchasing power parity-adjusted US dollars ($)) in the USA is approximately US $12,914 per person annually, US $7383 per person in Germany, and less than US $200 per person in most African countries [43]. Comparatively, while national healthcare expenditure as a share of the gross domestic product (GDP) is 18.3% in the USA, for example, it is less than 5% for most countries in Africa despite the very low GDP levels compared to the USA [44]. Strengthening public health budgets, improving healthcare infrastructure, and implementing robust policy frameworks are essential for enhancing programmatic improvements and ensuring adequate resources for TTR-CA prevention, diagnosis, and treatment.

The economic impact of TTR-CA in under-resourced environments is substantial, compounding the challenges faced by communities of African descent. The costs associated with diagnostic tests, treatments, hospitalizations, and long-term care place a significant burden on individuals, families, and healthcare systems. Moreover, the indirect costs, such as productivity losses and reduced quality of life, further contribute to the economic burden. Understanding the economic implications of TTR-CA is crucial to advocating for increased funding, developing cost-effective strategies, and implementing interventions that prioritize cost efficiency while delivering optimal care to individuals affected by TTR-CA.

Vulnerable populations in communities of African descent affected by TTR-CA often face a lack of social safety nets, worsening their health outcomes. The absence of support systems, such as disability benefits, social security programs, or health insurance coverage, can leave individuals without the financial means to access necessary medical interventions and supportive care. Establishing social safety nets that provide financial assistance, health insurance coverage, and support services can help protect vulnerable populations, alleviate the financial burden, and ensure equitable access to healthcare services for individuals at risk of or affected by TTR-CA.

Political will and supportive policy frameworks are crucial in addressing the burden of TTR-CA in communities of African descent. Governments need to prioritize healthcare investments, allocate adequate resources, and develop policies that support the prevention, early detection, and management of TTR-CA. Political commitment is essential to create a favorable environment for research, infrastructure development, and training programs. Additionally, policies that promote health equity, improve access to affordable healthcare services, and address socioeconomic determinants of health can help mitigate the disparities in TTR-CA outcomes among individuals of African descent.

The recognition and management of TTR-CA in communities of African descent are influenced by the complex interplay of various factors, including socioeconomic status, education, geography, cultural beliefs, and access to healthcare. These intersecting variables create unique challenges and disparities in disease recognition, diagnosis, treatment, and long-term management. It is crucial to adopt a holistic and multidimensional approach that considers the diverse factors that influence TTR-CA outcomes in these communities. This requires targeted interventions that address the specific needs, barriers, and cultural contexts of individuals of African descent.

## The way forward — a call to action

To address the uneven burden of TTR-CA in communities of African descent, a proactive approach is needed to build infrastructure and enhance capacity for an effective response (summarized in Table 1). This includes investing in healthcare infrastructure, establishing specialized centers of excellence, improving access to diagnostic tools and treatment options, and expanding training programs and career opportunities for healthcare professionals. Engaging community leaders, organizations, and patient advocacy groups is crucial for raising awareness, improving health literacy, and fostering a supportive environment for individuals affected by TTR-CA. The political leadership in low-resource economies must develop a clear vision for healthcare and proactively engage the international community in finding more sustainable ways to manage the global healthcare workforce to ensure global health equity.

**Table 1:** Overview of challenges and possible solutions to the burden of cardiac amyloidosis on the African continent

Challenge	Possible solutions
Lack of data	Establish registries to capture burden of disease including the following: • Cardiomyopathy registries • Autopsy series • Diastolic heart failure cohorts • Prospective treatment series
Lack of trained manpower	• Establish partnerships in training — for example, the Yale-Heart Institute of the Caribbean collaboration • Discourage brain drain through national policy changes • Encourage health workers to stay in their home countries by augmenting their paychecks
Access to diagnostic technology	Establish diagnostic programs that utilize affordable technology to diagnose cardiac amyloid at large scale • Strain echocardiography • Pyrophosphate nuclear scans • Genetic testing
Access to new treatments	• Discuss with pharmaceutical companies to subsidize the high cost of new treatments for cardiac amyloidosis • Additional funding could be sourced from philanthropic organizations
Lack of awareness	• Extensive health promotion and awareness campaigns using all available mass media tools including radio, television, social media, podcasts

Ensuring equitable access and care for vulnerable populations affected by TTR-CA requires the implementation of appropriate healthcare financing mechanisms. This can include expanding health insurance coverage, introducing targeted financial assistance programs, and developing innovative payment models that prioritize affordability and accessibility. Policymakers and healthcare stakeholders need to collaborate to design and implement financing mechanisms that protect and support individuals at risk of or affected by TTR-CA, particularly those from underserved communities with limited financial resources.

Big pharmaceutical companies have a pivotal role to play in ensuring inclusive access and health equity in the context of TTR-CA. Recognizing the economic challenges faced by low- and middle-income nations with predominantly Black populations, pharmaceutical companies should adopt geographical pricing strategies that balance profitability with affordability, social responsibility, and accessibility. Therapies for hTTR-CA are costly at present. For example, tafamidis is expensive (annual cost is approximately US $225,000), posing a considerable burden on uninsured or underinsured Black Americans, Africans in the diaspora, and West Africans who suffer the most from disease [45, 46]. It will be necessary for pharmaceutical companies and public health authorities to collaborate to ensure sick patients get access to this lifesaving therapy. Collaborative efforts between pharmaceutical companies, governments, and international organizations can help address the financial barriers and ensure the availability of affordable medications and treatments for TTR-CA in these communities. Additionally, partnerships between pharmaceutical companies and local healthcare providers can facilitate knowledge transfer, technology sharing, and capacity building to enhance diagnosis and treatment capabilities.

Building partnerships and collaborations between international centers of excellence and local institutions can facilitate knowledge exchange, technology transfer, and capacity building. Robust engagement of international centers of excellence in collaborative ventures with facilities serving populations of African descent is a promising strategy to improve access to TTR-CA diagnosis, treatment, and research in communities of African descent. A good model is the collaboration between the Heart Institute of the Caribbean (HIC) in Jamaica and Yale University School of Medicine to enhance the capacity for cardiovascular service delivery in the Caribbean [47]. HIC is actively engaged with the Yale Cardiovascular Amyloid group to develop methods to screen high-risk patients in the Caribbean for cardiac amyloidosis using both a rapid genetic testing approach and PYP imaging [48]. This project will create a prospective registry and will lay the foundations for further collaborative research involving therapies, clinical trials, and more widespread screening. Furthermore, the HIC/Yale collaboration is expected to include faculty and fellow exchanges, joint clinical conferences, program development, and enhancement, all of which will enhance local capacity for cardiovascular care delivery. HIC has also developed a thriving collaboration with the University of Pennsylvania that is focused on knowledge exchange with the goal of improving immediate delivery of heart failure care, with a long-term goal to create an advanced heart failure program in Jamaica, and increase engagement of University of Pennsylvania cardiologists and trainees at HIC [49].

These collaborations can leverage the expertise, resources, and technologies available at international centers to enhance local capacity and capabilities. Partnerships can involve knowledge exchange, training programs, research collaborations, and telemedicine initiatives. An example of such an international collaboration focused on education is the annual Nigerian Cardiovascular Symposium where invited Nigerian experts in the diaspora provided updates in cardiovascular medicine [50]. By working together, international centers of excellence and local institutions can bridge the gaps in resources, expertise, and infrastructure, ultimately improving access to high-quality care and advancing research in TTR-CA.

## Conclusions

Hereditary transthyretin cardiac amyloidosis is partly responsible for the high burden of heart failure in people of West African descent, given that the most common mutation of the transthyretin protein accountable for the disease occurs with a high allelic frequency in that population. Given that diagnostic tools are now more accessible and affordable and there is evidence-based treatment with mortality benefits for the condition, public health authorities and developmental organizations need to increase awareness of this important cause of heart failure as it is likely that screening and early diagnosis of this condition will lead to improvements in global health equity.

Addressing the uneven burden of cardiac amyloidosis, specifically TTR-CA, in communities of African descent requires a comprehensive and multidimensional approach. This includes addressing the challenges of limited data, deficit of trained workforce and resources, lack of infrastructure, affordability and access issues, health literacy, low public health budgets, weak infrastructure, political will, economic costs, lack of social safety nets, and the intersectionality of variables impacting disease recognition and management. Building infrastructure, enhancing capacity, implementing appropriate healthcare financing mechanisms, engaging big pharmaceutical companies, and fostering collaborative ventures with international centers of excellence are essential for promoting global health equity, improving access, and ensuring optimal care for individuals affected by TTR-CA in these communities.

## Data Availability

Not applicable.
